# Full-Digital Workflow for Fabricating a Custom-Made Direct Metal Laser Sintering (DMLS) Mandibular Implant: A Case Report

**DOI:** 10.3390/ijerph17082693

**Published:** 2020-04-14

**Authors:** Francesco Grecchi, Piero Antonio Zecca, Aldo Macchi, Alessandro Mangano, Federica Riva, Emma Grecchi, Carlo Mangano

**Affiliations:** 1 Department of Maxillofacial Surgery, IRCCS Istituto Ortopedico Galeazzi, Via Galeazzi 4, 20161 Milan, Italy; dr.grecchi@tiscali.it (F.G.); federicariva85@gmail.com (F.R.); 2 Department of Medicine and Surgery, Dental School, University of Varese, Via Piatti 10, 21100 Varese, Italy; pieroantonio@gmail.com (P.A.Z.); aldo.macchi@uninsubria.it (A.M.); 3 Private Practice, 22015 Gravedona ed Uniti, Italy; 4 Department of Oral Surgery, IRCCS Fondazione Ca’ Granda, Ospedale Maggiore Policlinico, Via Sforza 35, 20122 Milan, Italy; emma.grecchi2@gmail.com; 5 Department of Dental Sciences, University Vita Salute San Raffaele, Via Olgettina 60, 20132 Milan, Italy; camangan@gmail.com

**Keywords:** CAD/CAM, titanium mesh, mandibular resection

## Abstract

Direct Laser Metal Sintering (DLMS) is an additive manufacturing (AM) technique that is capable of manufacturing metal parts according to a three-dimensional (3D) design made using computer-assisted-design (CAD) software, thanks to a powerful laser beam that melts selectively micro-powder layers, one on top of the other, until the desired object is generated. With DMLS, it is now possible to fabricate custom-made titanium implants for oral and maxillofacial applications. We present the case of a 67-year-old woman diagnosed with a squamous cell carcinoma of the mandible. The patient underwent subtotal mandibular resection; conventional reconstruction procedures failed to rehabilitate the function of the mandible. A prosthesis replacing the resected mandible was designed and fabricated using a digital workflow. The extensive bone defect was rehabilitated with a prosthesis replacing the mandibular bone and supporting a morse-taper dental prosthesis. The masticatory function was reestablished.

## 1. Background

Mandibular discontinuity, caused by tumor ablation or trauma, is typically reconstructed using a vascularized or non-vascularized bone graft [1]. However, block bones cannot reproduce the natural curve and configuration of the defected mandible [1]. In the 1990s, several oral and maxillofacial surgeons attempted to achieve more accurate mandibular reconstruction with a custom-made Titanium (Ti) mesh tray and particulate cancellous bone and marrow (PCBM) [2].

Gold standards of treatment for reconstructing segmental defects after resective surgery include advanced microsurgery with fibula-free flaps with costochondral rib and iliac bone grafts [3].

The digital revolution is changing the way we work [4]. The introduction of new technologies such as additive manufacturing (AM) techniques is improving the rehabilitation possibilities for patients, and is leading to the development of more precise tools for clinicians [5].

Ranging from cone-beam computer tomography (CBCT), computer-assisted-design (CAD) software, intraoral scanners, and 3D printing machine to AM techniques, all these new technologies have improved the diagnosis process and revolutionized the workflow in oral and maxillofacial surgery [6,7].

In particular, Direct Laser Metal Sintering (DLMS) is an additive manufacturing (AM) technique that is capable of manufacturing metal parts according to a three-dimensional (3D) design made using computer-assisted-design (CAD) software [8]. The CAD project is subsequently sent to a machine that, thanks to a powerful laser beam, melts selectively micropowder layers, one on top of the other, until the desired object is generated [8].

Additive manufacturing technologies and DMLS make it possible to fabricate not only standard dental implants using titanium alloy, but also custom-made devices or implants, tailored to the specific needs of different patients, such as meshes for bone regeneration, blades and root analogue implants [9].

The possibility of fabricating custom-made implants opens interesting perspectives in oral and maxillofacial surgery [10]. Customized meshes make it possible for the surgeon to perfectly fit a specific device to a patient’s defects, shortening the duration of the operation and, therefore, reducing the risk of postoperative graft infection [10]. Similarly, thanks to DMLS, it is possible to produce subperiosteal implants for fixed prosthetic rehabilitation of the posterior mandible in patients with reduced height or bone thickness who do not want to undergo regenerative bone surgical procedures [11]. Nevertheless, it is in maxillofacial surgery that the use of DMLS custom-made implants can find an ideal application, where patients who have suffered extensive damage to the jaws due to traumatic injury or cancer resection must be rehabilitated [11].

We present the case of a patient rehabilitated with a custom-made DMLS mandible after subtotal mandibular resection due to a squamous cell carcinoma.

## 2. Case Presentation

A 67-year-old woman was referred to the Department of Maxillofacial Surgery of the Galeazzi Orthopedic Institute five years after the diagnosis and treatment of a mandibular carcinoma. The clinical history of the patient was rather complex.

**Figure 1:**
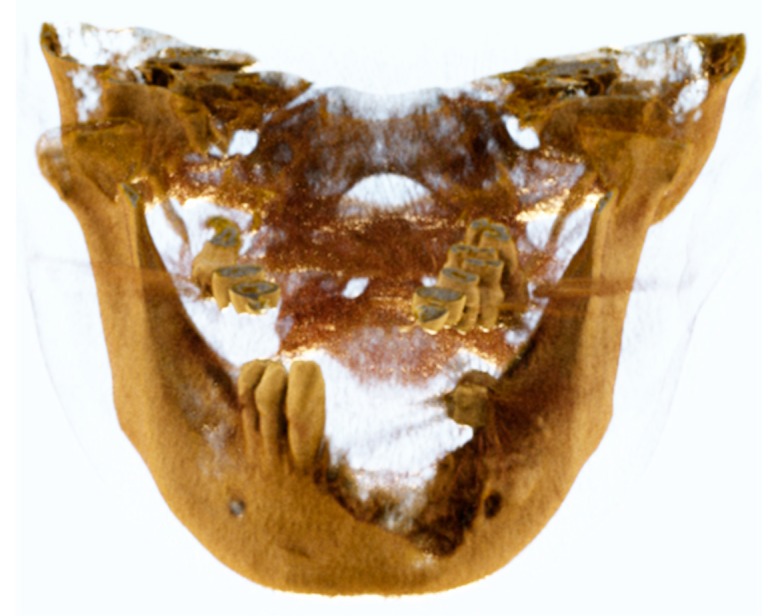
Computerized tomography (CT) scan that showed the presence of a tumor mass in the anterior region of the mandible.

**Figure 2:**
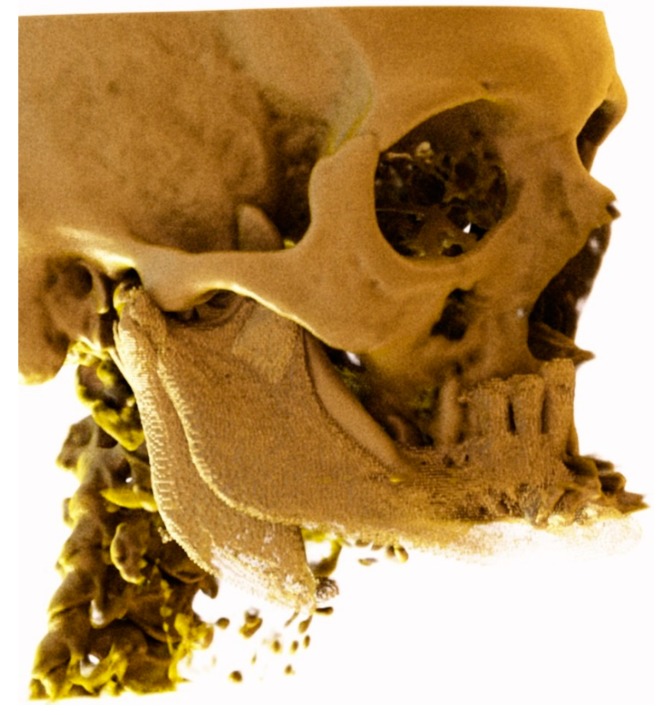
After the resective surgery of this lesion, the patient presented tumor recurrence within a few months. For this reason, she underwent two additional resective surgeries to remove the tumor. In the end, the patient underwent the complete removal of the body of the jaw.

In 2012, after a dental visit due to periodontal disease of frontal inferior teeth, the patient underwent a computerized tomography (CT) scan that revealed the presence of an osteolytic lesion in this site (Figure 1). A preoperative histopathological examination revealed an intraosseous squamous cell carcinoma. The medical history data of the patient revealed no systemic diseases or smoking habits. The patient was treated with surgical removal of the lesion through subtotal mandibular resection, associated with a temporary tracheostomy, bilateral lymph node neck dissection and fibula free flap reconstruction (Figure 2). After flap failure, a second fibula free flap was performed, but this again failed. Seven months later, a spontaneous internal jugular vein and external carotid artery laceration was treated by vessel ligation. The pectoralis flap was then used to supply soft tissue defects. Oncological follow-up observed a recurrence nineteen months after the first surgery. Multiple cycles of chemotherapy and monoclonal therapy were then administered. Radiotherapy was refused by the patient. 

The first clinical evaluation in our department revealed an important deformity in the inferior third of the face associated with swallowing and language deficiency caused by tongue hypomobility and lower lip paralysis, incontinence, and incompetence. Feeding was possible only with percutaneous endoscopic gastrotomy. Further reconstructive treatment with free flaps was contraindicated. Therefore, it was decided to reconstruct the mandible with a CAD/CAM technique and with a custom-made DMLS titanium implant. The patient was informed about the procedure in full detail and signed an informed consent form dedicated to this therapy. The study protocol was approved by the Ethics Committee of the University of Insubria (n’ 826, 10/08/2013). All procedures performed complied with the World Medical Organization’s Declaration of Helsinki.

### 2.1. Data Acquisition

The patient underwent cone-beam computed tomography (CBCT) (CS 9300^®^, Carestream Dental, Atlanta, GA, USA) for the acquisition of 3D bone volume. Specific fields of view were selected (17 × 13.5 cm, with a slice thickness of 60 to 200 μm) to obtain high-resolution digital imaging.

The digital imaging and communication in medicine (DICOM) data were then transferred to a software for 3D reconstruction (Mimics^®^, Materialise, Leuven, Belgium) for segmentation. The segmentation process allowed us to identify and define the appropriate anatomical structures by separating them, with the final purpose of clearly visualizing all components and obtaining a 3D virtual model of the residual bone portions. The segmentation process consisted of two phases: one automatic and one manual. The first phase used the different density values (grey level units) of the tissues to delineate their boundaries. In this first phase, the segmentation objects were defined on a lower threshold, and they contained all the pixels in the images with a value higher or equal to the threshold value. However, the final segmentation was adjusted manually, with the operator being required to delineate and control the anatomical structures slice by slice.

### 2.2. CAD 3D Modelling

In reconstructing any defect, not only the pathology, but also the surrounding anatomical areas must be printed for proper 3D orientation. Using the Meshnoturb command of the Rhinoceros software (Rinhoceros^®^, Robert Mc Nell & Associates, Seattle, WA, USA), the 3D models were converted into closed polysurfaces with a mean value of about 20,000 surfaces. These were then exported as Initial Graphics Exchange Specification files to Comsol Multiphysics v.4.2.a software (Comsol INC, Burlington, MA, USA). The computer used for mechanical computing was a Hewlett Packard workstation (Intel core i7, 96 GB). This utilized 350,000 tetrahedral mesh elements and required a maximum of 10 min for meshing and a minimum of 5 min for analysis. Printing was done using Poly-L-lactide, and the properties evaluated for this material included Young’s modulus (Es = 3.3 GPa), Poisson’s ratio (m = 0.3), and density (q = 1.3 g/cm^3^). The model was subjected to compression experiments which included displacement of the top surface (maximum engineering strain, emax = 1%), fixation of the bottom surface, and symmetry of the remaining surfaces. Other parameters evaluated included the true reaction stress at the bottom surface, von Mises stress, displacement field, and strain energy density. The Young’s modulus of porous material was also calculated as the ratio between true reaction stress at the bottom surface and axial engineering strain.

### 2.3. Direct Metal Laser Sintering (DMLS)

The custom-made implant was fabricated using DMLS. A Titanium alloy grade 5 (Ti 6Al-4v) was used with micropowder grain with a size of 30–50 µm. A high-powered ytterbium fiber laser, which uses a wavelength of 1060 to 1100 nm (DMP Dental100, Biotec-BTK, Povolaro di Dueville-Vicenza, Italy), was used to melt metallic micropowders, layer by layer, into a predetermined shape based on CAD data. First, a layer of powder was deposited over the building platform, which acted as a base for selective deposition of layers of powder. Each cross-section of the model was scanned by the laser, which then selectively deposited and melted the powder accordingly. This process continued until the entire implant was fabricated. The construction of the device was carried out in a tightly controlled atmosphere to prevent contamination of the material, especially by expelled debris, and avoiding exposure to nitrogen and oxygen. 

### 2.4. Post-Production

In order to remove any residual, nonadherent titanium particles, the implant was first sonicated for 5 min in distilled water at 25 °C, immersed in sodium hydroxide (20 g/L^−1^) and hydrogen peroxide (20 g/L^−1^) at 80 °C for 30 min, and finally, sonicated for 5 min in distilled water. Acid etching was carried out by immersion of the samples in a mixture of 50% oxalic acid and 50% maleic acid at 80 °C for 45 min, followed by washing for 5 min in distilled water in a sonic bath. A stress-relieving postprocessing heat treatment was performed in an argon atmosphere for 2 h at a temperature of 600 °C. The direct laser preparation provided an implant surface with a roughness with a mean (± standard deviation) of the absolute values of all profile points, the root-mean-square of the values of all points, the average value of the absolute heights of the five highest peaks, and the depths of the five deepest valleys of 66.8 ± 6.6 mm, 77.6 ± 11.1 mm, and 358.3 ± 101.9 mm, as previously described in the literature [8].

### 2.5. Surgery

**Figure 3:**
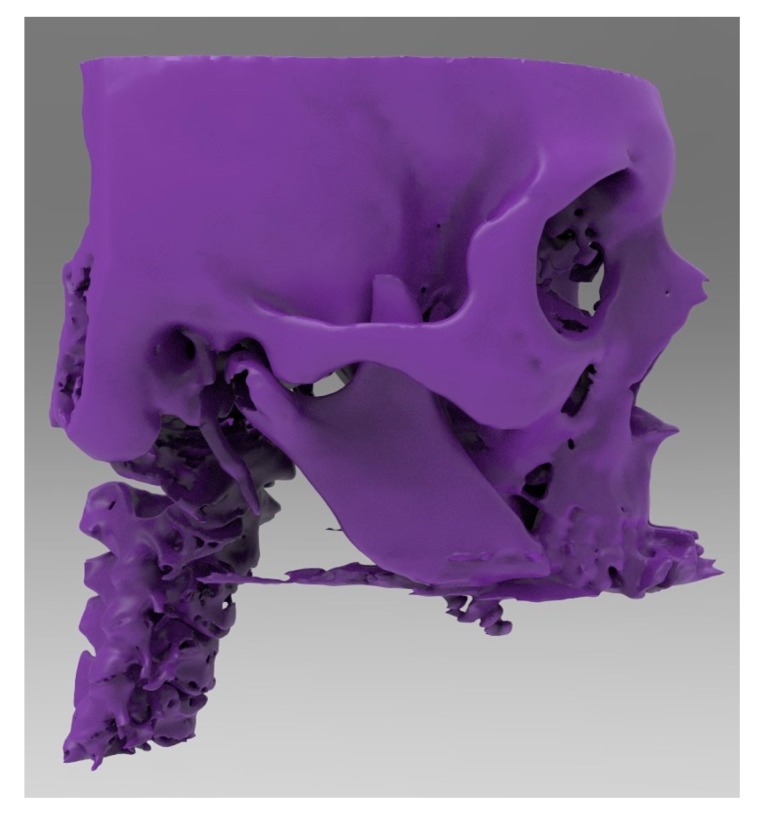
3D reconstruction of the mandibular branches displaced due to the temporal contraction.

This case was challenging because the patient underwent several surgeries that led to an important functional and esthetic deficiency. The residual portions of the mandible were rotated and displaced due to the temporal muscle traction issues shown in the CT scan (Figure 3), making it impossible to use them in this position for the custom-made prosthesis project.

**Figure 4:**
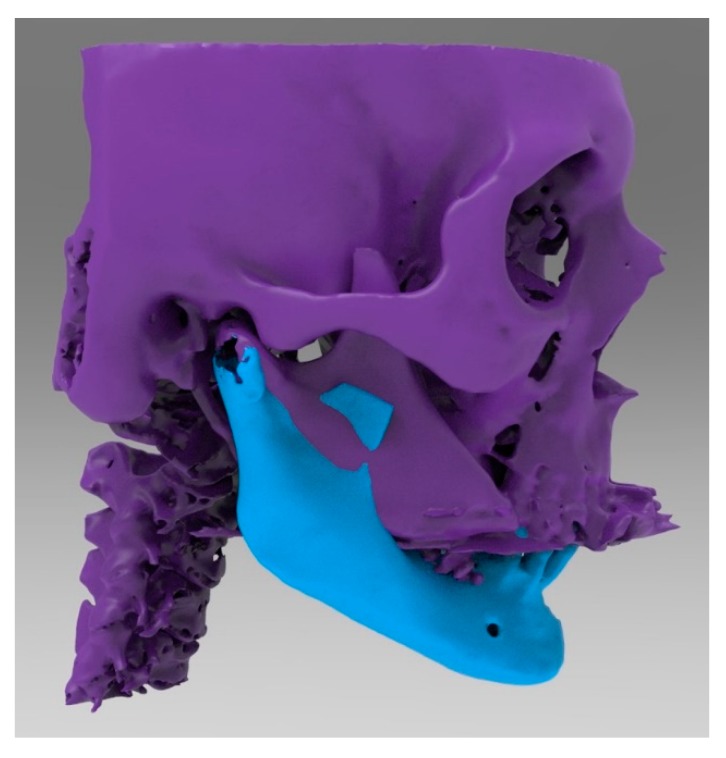
The preintervention computerized tomography (CT) was superimposed with the post CT in order to determine the original position of the mandible.

**Figure 5:**
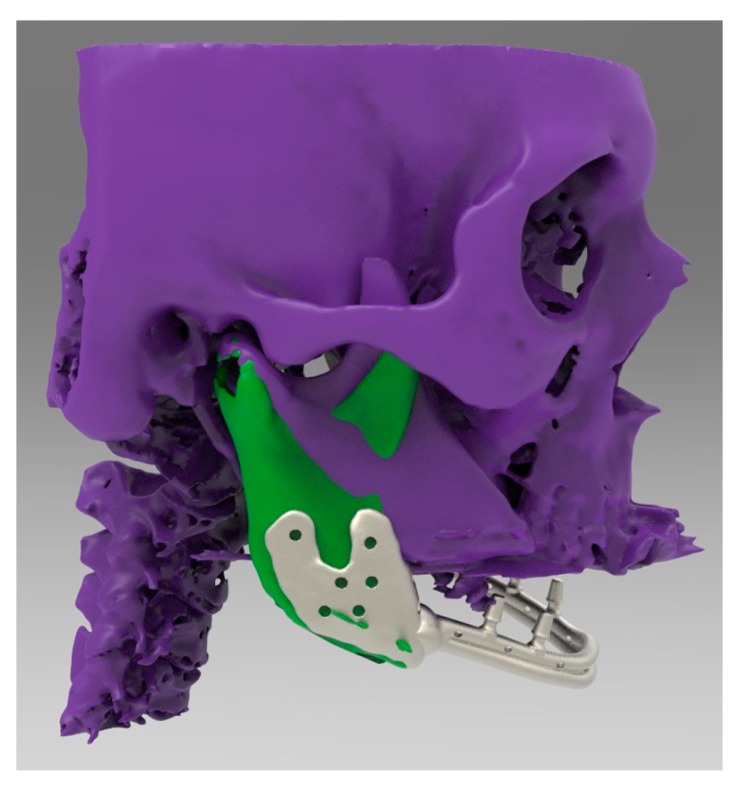
Lateral view of the mandibular body that was shaped and connected to the residual mandibular branches.

**Figure 6:**
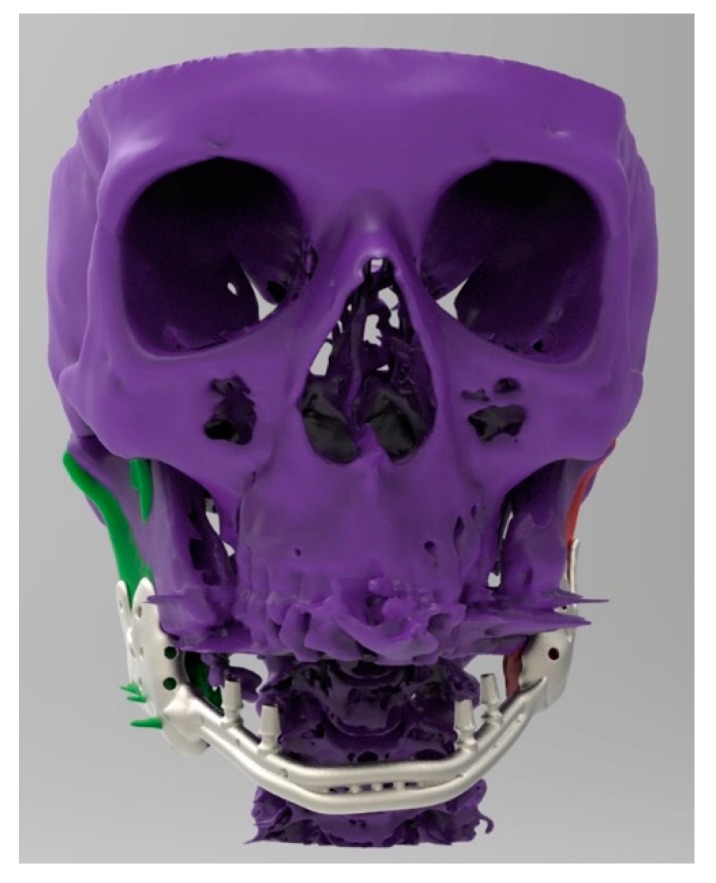
Frontal view.

**Figure 7:**
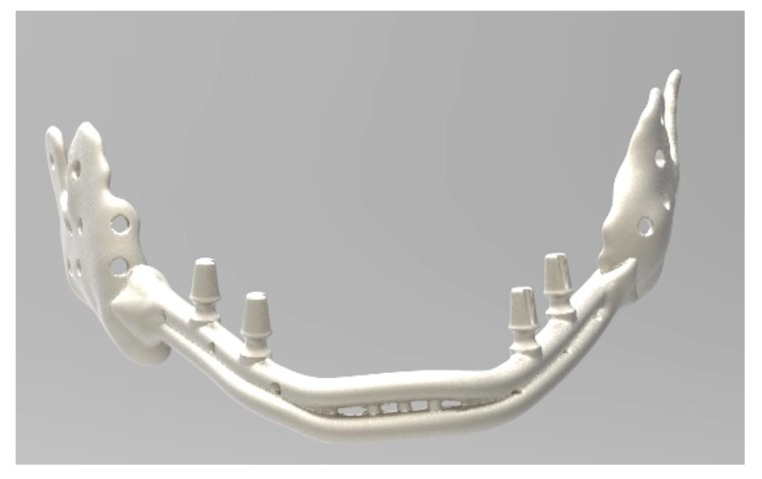
Frontal view of computer-aided-design (CAD) design (.stl file).

**Figure 8:**
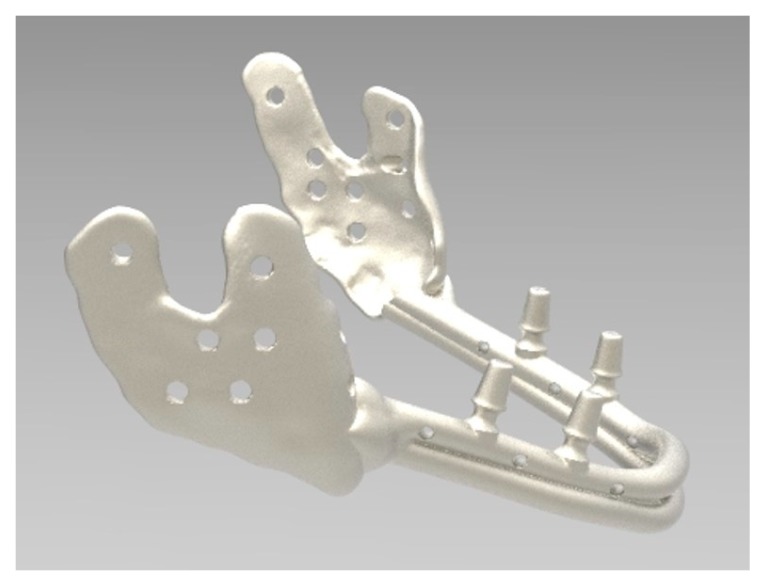
Lateral view of the same computer-aided-design (CAD) design.

**Figure 9:**
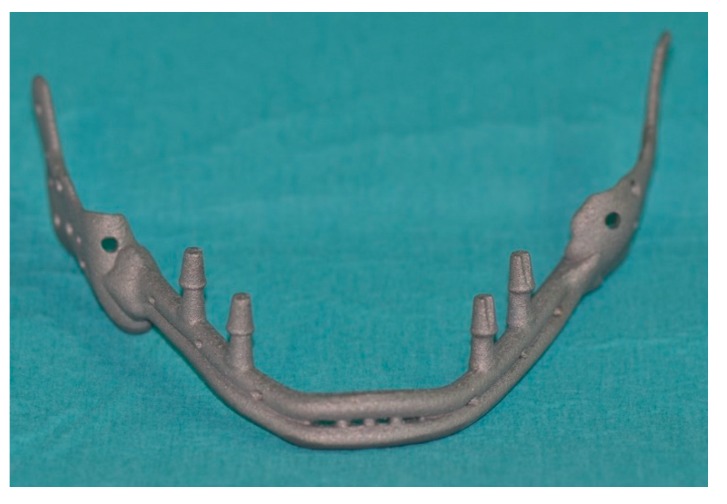
The mandibular prosthesis produced by direct metal laser forming (DLMF) technology.

The preintervention CT was superimposed with the postoperative CT to determine the correct position of the residual mandible. Afterward, the postoperative mandibular ramus was superimposed on the preoperative mandible in order to place the mandibular ramus in the desired position, compared to the one before the resective surgery (Figure 4). From this new anatomy, a mandibular body prosthesis was shaped with the following characteristics:

- The prosthesis reflected the original anatomy of the jaw.
- The prosthesis made it possible to suspend soft tissue by holes in the anterior portion.
- The prosthesis had the possibility of osteosynthesis by screws in the posterior portions.
- The mandibular prosthesis was designed with the morse-taper connections for the overdenture (Figure 5, Figure 6, Figure 7, Figure 8 and Figure 9).

**Figure 10:**
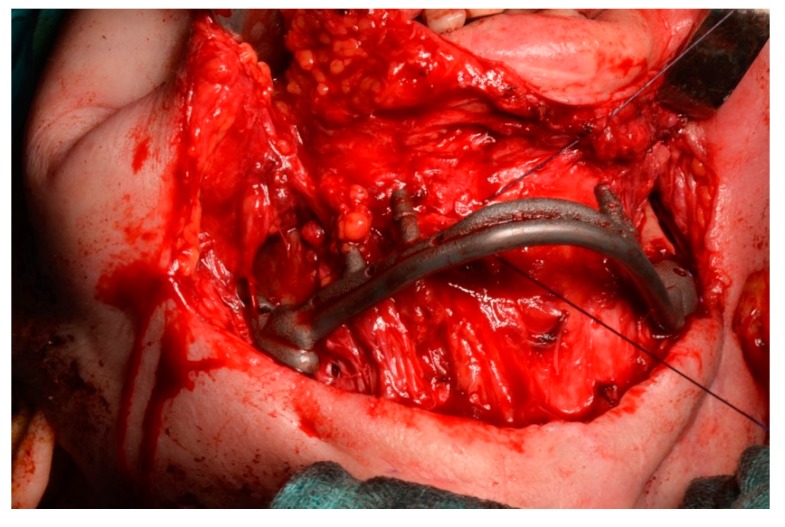
The mandibular prosthesis connected to the residual branches by osteosynthesis screws.

**Figure 11:**
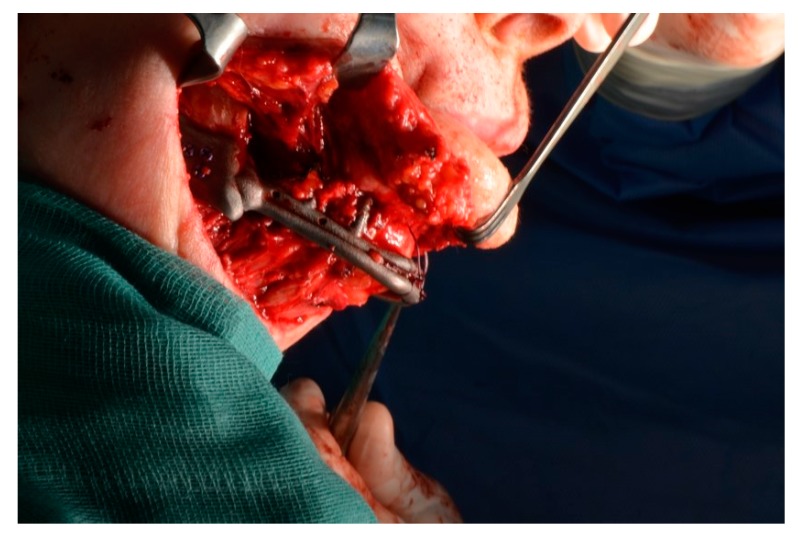
Lateral view.

**Figure 12:**
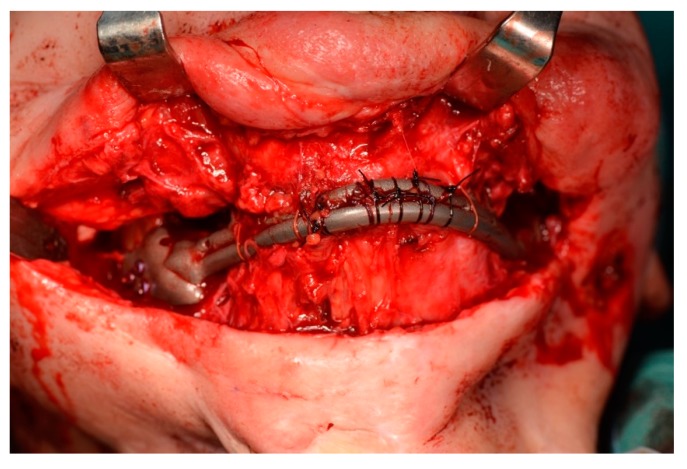
Suspension of soft tissue and overioid muscles to the anterior portion of the prosthesis.

Once the custom-made jaw prosthesis was created, reconstructive surgery was performed under general anesthesia through these steps:

- A temporary tracheostomy to protect the airway was performed.
- A wide cutaneous neck incision was made to achieve the residual portions of the mandible and prepare the surgical field to receive the prosthesis.
- Bilateral coronoidotomy was performed to release the ramus. The structures were rotated and repositioned.
- The jaw prosthesis placement and fixation was completed using screws (Tekka^®^, Pesaro, Italy) of 2 mm Ø and 7 to 9 mm length to attach them to the mandibular ramus.
- Soft tissue was suspended to the anterior portion of the prosthesis.
- An intraoral mucosa incision was made to expose the stumps for the following dental rehabilitation.
- The cutaneous access was then sutured. (Figure 10, Figure 11 and Figure 12)

## 3. Results

**Figure 13:**
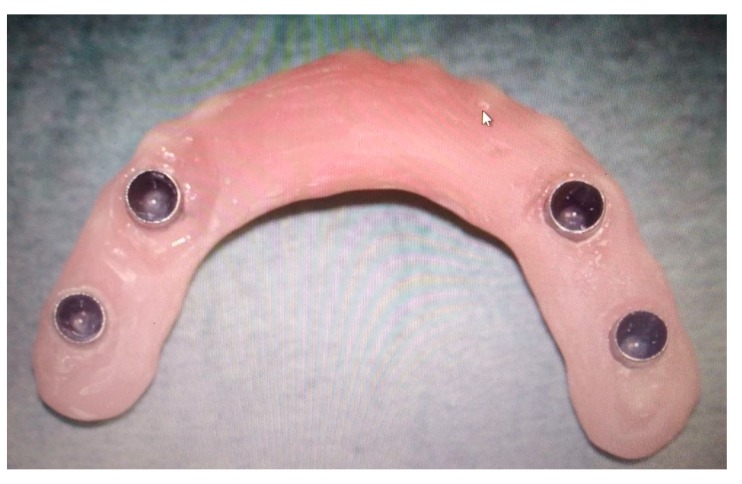
The removable prosthesis with morse-taper connection to the abutments of the mandibular prosthesis.

**Figure 14:**
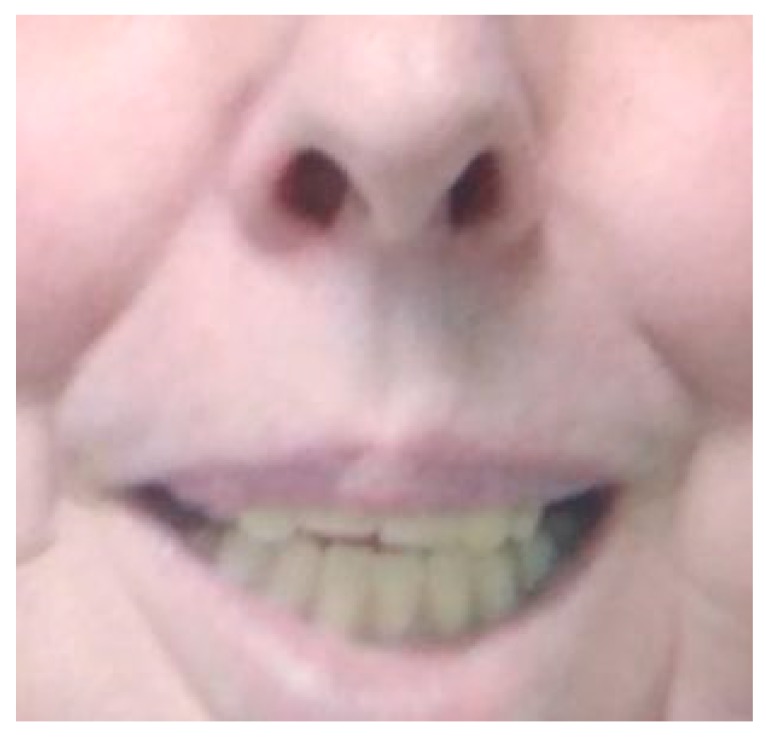
Intraoral view of rehabilitation.

**Figure 15:**
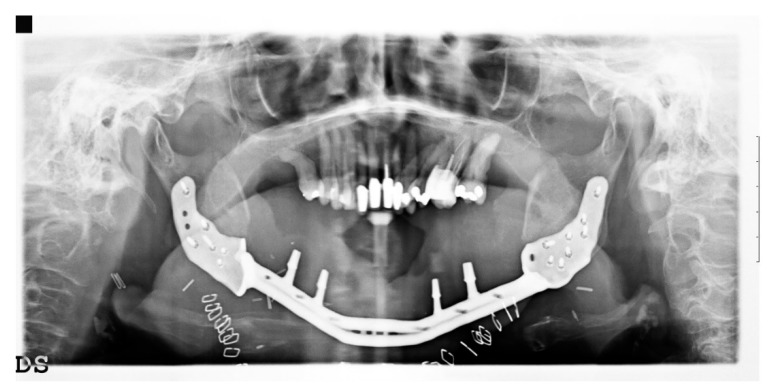
Orthopantomography two months after surgery.

**Figure 16:**
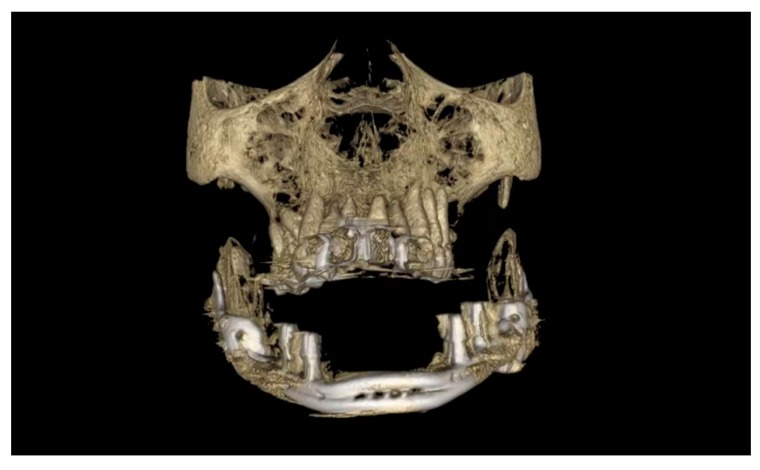
Computerized tomography (CT) after 2 years.

**Figure 17:**
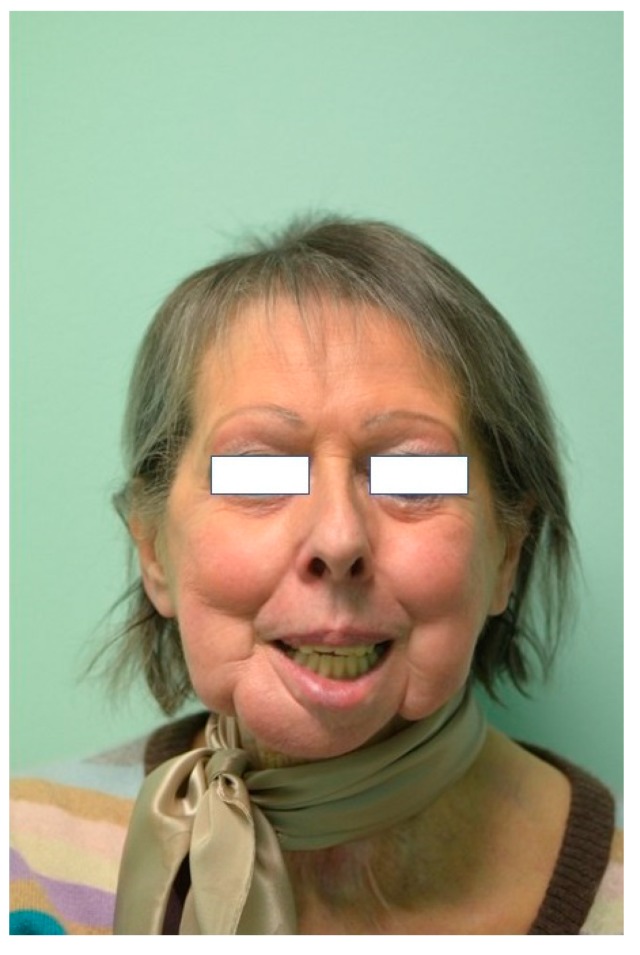
Frontal view picture at the 2-year follow-up.

One week after surgery, the patient could leave the hospital presenting no complications. Prosthetic restoration (Figure 13 and Figure 14) was delivered in the same structure, two months after surgery, after the resolution with laser therapy of an oral mucositis affecting the tissues around one of the integral abutments. The two-month postoperative orthopantomography showed a perfect integration of the mandibular prosthesis (Figure 15). Ten months after surgery, two areas of cutaneous exposure of the prosthesis were observed; local cutaneous flaps were used to fix them. The custom-made implant showed perfect integration and radiological stability, with no further signs of exposure two years after the surgery. The functional and aesthetic improvement was excellent, as perceived by the patient. The 24-month follow-up (Figure 16 and Figure 17) revealed a good general health status and no sign of cancer disease. 

## 4. Discussion

The DMLS technique presents several advantages [8]. It can reproduce parts with high geometrical complexity [8]. It does not require expensive molds as it can be directly printed from a CAD model. The production steps are minimized, resulting in low labor and tooling costs. It is highly flexible, and changes in design can be quickly made. It generates very little waste and can utilize hard materials that are otherwise difficult to process due to their high melting points. The DMLS titanium prosthesis was designed and fabricated with a morse-taper connection that allowed the placement of a removable total prosthesis [8].

Reconstruction of the mandible is always a challenging clinical task due to the complexity of anatomy, function, and aesthetic role [12]. In particular, restoring form and function plays a pivotal role [13].

CAD/CAM technologies are a powerful tool for surgeons, allowing careful planning of the final outcome [14]. In our case, the preoperative planning results were accurate and reliable, allowing us to reduce the duration of the surgery.

The gold standard for the reconstruction of critical-sized mandibular defects employs autogenous bone [1]; however, patient, surgeon and donor-site limitations may preclude candidacy for this operation [1,2]. In our experience, DMLS could overcome some of these limitations, giving rise to predictable restorations of anatomical defects [15,16].

Novel, three-dimensional, surgical simulation software has rendered reconstructive surgery much more reliable than it was before [17]. During the surgery, we were able to easily insert the mandibular prosthesis due to careful presurgical planning.

Computer-aided navigation software via three-dimensional simulation of the resected bone allows the surgeon to visualize the anatomical features in considerable detail, and to plan the reconstructive surgery appropriately [6,10]. Moreover, precise, intraoperative, three-dimensional anatomical orientation is possible. This greatly aids in the definition of the subsequent position of the prosthesis [18]. The method used allowed us to fabricate and install a precise mandibular prosthesis. The anatomy and function of our patient was fully restored. We observed a complication two months after the surgery due to cutaneous exposure of the prosthesis; this was solved with the local cutaneous flap. The oral mucositis was treated with laser therapy to prevent antibiotic resistance [19]. We preferred not to administer antibiotics to prevent the occurrence of possible bacterial resistance [20].

Furthermore, according to Tarsitano et al., [21] the use of a 3D designed and fabricated prosthesis could provide advantages in terms of biomechanical stress. The bending of an osteosynthesis plate can introduce unpredictable stresses into the reconstructed site, resulting in the risk of failure [22].

## 5. Conclusions

CAD-CAM surgery can be considered a useful tool for improving the quality of reconstructions and reducing the need for secondary revision procedures such as flap repositioning for restoring a proper occlusion. Moreover, guided surgery has the potential not only to reduce surgical time, but also, to reduce prosthesis-related complications. Within the limits of this study, DMLS technology was demonstrated to be a useful and predictable way to produce prostheses for major reconstructive surgery.

## Abbreviations

DMLS	Direct Metal Laser Sintering
AM	Additive Manufacturing
CAD/CAM	Computer-aided design/Computer-aided technology
CBCT	Cone-beam computed tomography
CT	Computerized tomography
PCBM	Particulate cancellous bone and marrow
